# MET receptor variant R970C favors calpain-dependent generation of a fragment promoting epithelial cell scattering

**DOI:** 10.18632/oncotarget.14499

**Published:** 2017-01-04

**Authors:** Rémi Montagne, Anne Baranzelli, Ghaffar Muharram, Leroy Catherine, Marie Lesaffre, Audrey Vinchent, Zoulika Kherrouche, Elisabeth Werkmeister, Alexis B Cortot, David Tulasne

**Affiliations:** ^1^ University Lille, CNRS, Institut Pasteur de Lille, UMR 8161-M3T-Mechanisms of Tumorigenesis and Targeted Therapies, F-59000 Lille, France; ^2^ University Lille, CHU Lille, Thoracic Oncology Department, F-59000 Lille, France; ^3^ University Lille, CNRS, Inserm, CHU Lille, Institut Pasteur de Lille, U1019-UMR 8204-CIIL-Center for Infection and Immunity of Lille, F-59000 Lille, France; ^4^ BioImaging Center Lille, Lille 59021, France

**Keywords:** lung cancer, MET, receptor tyrosine kinase, hepatocyte growth factor/scatter factor, proteolytic cleavages

## Abstract

The receptor tyrosine kinase MET and its ligand, the hepatocyte growth factor, are essential to embryonic development, whereas deregulation of MET signaling is associated with tumorigenesis leading to various cancers, including lung carcinoma. Mutations in the MET kinase domain lead to constitutive kinase activity and are associated with tumorigenesis. In lung cancer, however, some mutations are found in the juxtamembrane domain, and their functional consequences are unknown. Because the juxtamembrane domain of MET is targeted by several proteolytic cleavages, involved in its degradation during cell death or under steady-state conditions, we evaluated the influence of these mutations on the MET proteolytic cleavages. In stably transfected epithelial cells expressing MET, the juxtamembrane mutations R970C, P991S, and T992I were found not to modify the known caspase or presenilin-dependent regulated intramembrane proteolysis. Yet when overexpressed, the R970C variant caused generation of an as yet undescribed 45-kDa fragment (p45 MET). This fragment was found in the confluent lung cancer cell line NCI-H1437 carrying the R970C mutation and at a lesser extent in cell lines expressing WT MET, suggesting that R970C mutation favors this cleavage. Generation of p45 MET required the activity of the calpain proteases, confirming the involvement of proteolysis. Ectopic expression of reconstituted p45 MET in epithelial cell lines favored cell scattering and invasion indicating active role of this fragment in HGF/SF induced responses. Hence, although the juxtamembrane mutations of MET do not affect its known proteolytic cleavages, the R970C MET variant favors calpain dependent proteolytic cleavage in lung cancer cells.

## INTRODUCTION

MET is a receptor tyrosine kinase (RTK) expressed predominantly in cells of epithelial origin. It is activated by its stromal ligand, the hepatocyte growth factor/scatter factor (HGF/SF) [1]. HGF/SF and MET are essential to embryonic development, since knockout of either one affects epithelial organ, placenta, muscle, and neuron formation [2–5]. Conditional knockout of MET in the lung inhibits alveolar development, a possible consequence of decreased alveolar epithelial cell (AECII) proliferation and survival [6]. In adults, the HGF/SF-MET pair is involved in physiological processes such as epidermal healing and liver regeneration [7–9].

Upon ligand binding and subsequent MET dimerization, several tyrosine residues in the intracellular portion become phosphorylated, allowing recruitment of cytoplasmic proteins involved in activating multiple intracellular signaling pathways [10, 11]. Downregulation of the MET receptor is an essential negative regulatory mechanism preventing receptor oversignaling. Besides the well-known ligand-dependent degradation of MET, notably involving lysosomal degradation of the ubiquitinated receptor, we have demonstrated that MET undergoes several proteolytic cleavages also involved in downregulation of the receptor. Under apoptotic conditions, MET is cleaved by caspases within the juxtamembrane and C-terminal domains. These cleavages separate the extracellular ligand-binding domain from the intracellular kinase domain [12–15], inactivate the receptor, and create a 40-kDa intracellular fragment (p40 MET^caspase^) that can amplify cell death by promoting mitochondrial permeabilization [16]. During calcium stress-induced necrosis, we recently showed that MET undergoes cleavages by calpains 1 and 2. This cleavage leads to the generation of an about 40 kDa intracellular fragment named p40 MET^calpain^. This fragment is unable to favor neither necrosis nor apoptosis [17]. Thus, while p40 MET^caspase^ and p40 MET^calpain^ fragments are quite similar, only p40 MET^caspase^ is an active fragment promoting cell death. Under steady-state conditions, MET is processed by Presenilin-Regulated Intramembrane Proteolysis (PS-RIP). This proteolytic process involves a first cleavage by membrane ADAM metalloproteases, leading to generation of a membrane-anchored p55 MET fragment (or MET-C Terminal Fragment, MET-CTF) also referred as MET-EC^-^ (MET lacking the ectodomain). Ectopic expression of this membrane-anchored fragment is able to promote cellular invasion and tumor growth in experimental models [18]. However, it is effectively degraded by the lysosome [19]. This fragment can be also further cleaved by γ-secretase, yielding a p50 MET fragment (or MET-Intra Cellular Domain, MET-ICD) degraded by the proteasome [20]. These cleavages contribute to decreasing the half-life of the receptor and to preventing its accumulation in the membrane through generation of labile intracellular fragments.

Aberrant MET and HGF/SF signaling is involved in promoting tumorigenesis and metastasis. In a significant number of human cancers, HGF/SF and MET are overexpressed [1]. About half of all non-small-cell lung cancers (NSCLCs) overexpress MET. This overexpression is associated with poor prognosis [21–23]. In addition, in about 5–20% of the NSCLCs displaying a mutated Epidermal Growth Factor Receptor (EGFR) gene, acquired resistance to EGFR inhibitors involves amplification of the *MET* gene, leading to its strong overexpression and activation [24]. In a variety of cancers, furthermore, many MET mutations have also been discovered.

In renal cancers, MET mutations are located mainly in the kinase domain, causing constitutive kinase activity. In lung cancers, MET mutations are found in 3 to 10% of cases according to the ethnic origin [25], but in contrast to those found in renal cancers, they mainly affect the juxtamembrane domain. The first group of mutations impairs the acceptor and donor splicing sites of the exon 14 leading to exon skipping and deletion of a large part of the juxtamembrane domain. These mutations were found in about 3 % of the NSCLC and include various punctual mutations and deletions all targeting the splicing sites. Deletion of the juxtamembrane domain favors receptor activation by its ligand, since this domain displays several negative regulatory sites [26]. The second group of mutations affecting the juxtamembrane domain comprises punctual mutations inducing amino acids substitution within the domain. These mutations include the R970C, P991S, and T992I substitutions (respectively R988C, P1009S, and T1010I in the long isoform of MET) with for instance about 1% of the patients for the R970C variant [25]. In contrast to mutations affecting the splicing sites or the kinase domain, it is unknown how this amino acid substitutions within the juxtamembrane domain affect MET functionally. While studies have demonstrated that they favor the growth of experimental tumors, they do not cause MET kinase activation [27–29]. In addition, although these mutations were initially identified in lung tumors, recent studies have shown that they can be germline that might correspond to polymorphisms [25, 29–31]. Yet in the mouse strain SWRJ, the R968C MET variant, corresponding to the human R970C variant, favors the development of lung tumors, suggesting that it modifies MET activity through an unknown mechanism [32]. It is thus important to understand the functional consequences of these MET mutations.

Because caspases and γ-secretase MET cleavages target the juxtamembrane domain, we have sought to evaluate how the juxtamembrane mutations found in lung tumors affect proteolysis. We demonstrate that the R970C, P991S, and T992I variants do not affect the known proteolytic cleavages induced during cell death and by the PS-RIP process. Yet we further show that the R970C mutation favors generation of a novel 45-kDa fragment (p45 MET). In lung cancer cell lines carrying the R970C mutation, we show that generation of this fragment is regulated by cell density and involves proteolytic cleavage by calpains. Furthermore, expression of the reconstituted fragment in epithelial cells favors scattering, motility and invasion induced by HGF/SF. Our results thus demonstrate that a juxtamembrane mutation of MET can promote its proteolytic cleavage in lung cancer cells leading to generation of an active fragment.

## RESULTS

### Juxtamembrane mutations do not modify the known proteolytic cleavages of MET

**Figure 1:**
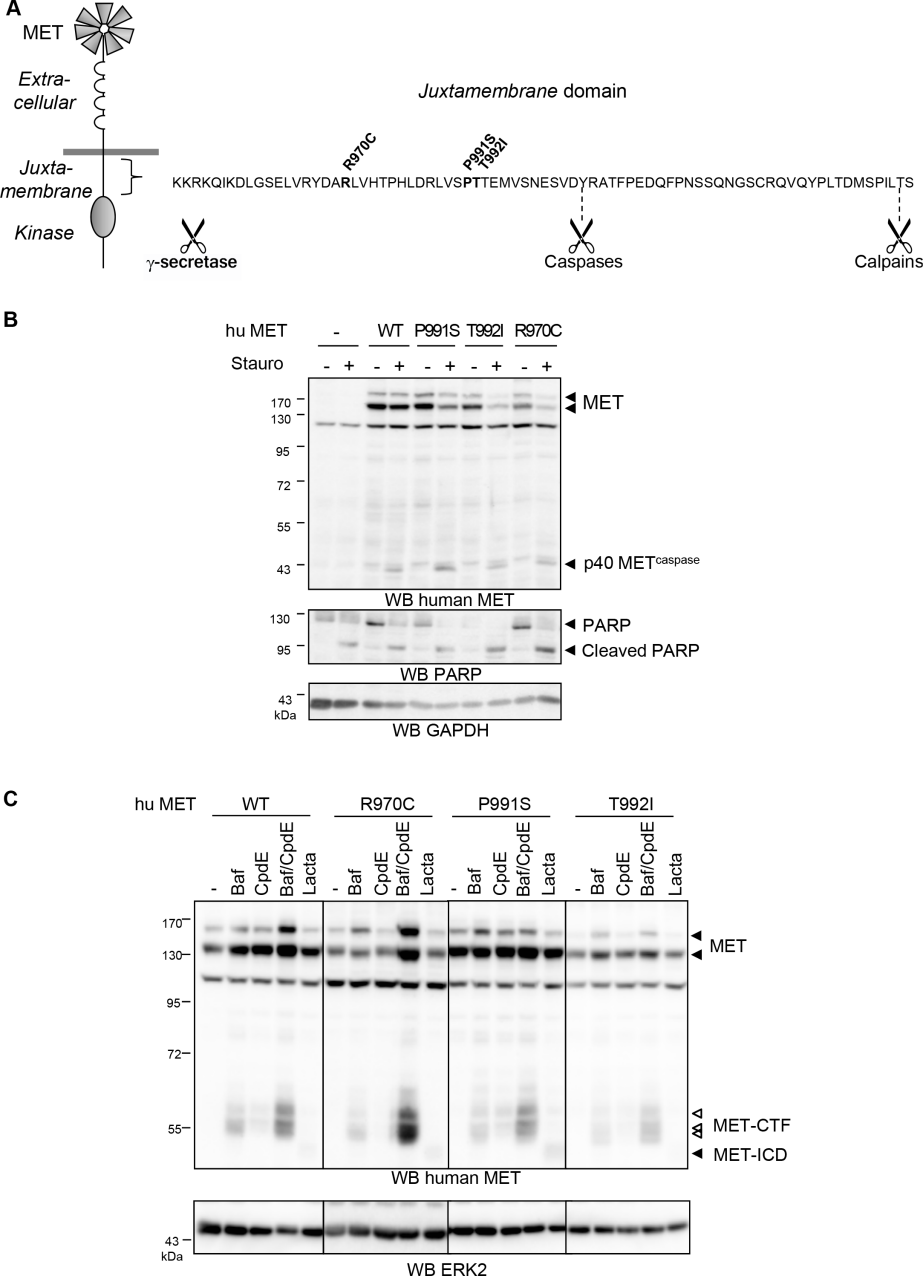
Effects of MET juxtamembrane mutations on caspases and secretases cleavages (**A**) Schematic representation of MET receptor with the extracellular, juxtamembrane and kinase domains and amino acids sequence of the juxtamembrane domain with positions of the R970C, P991S, and T992I mutations and cleavage sites by γ-secretase, caspases during apoptosis and calcium regulated calpains during necrosis. (**B**) MDCK cells stably expressing WT, P991S, T992I, or R970C human MET (hu MET) were treated for 8 h with staurosporin (Stauro; 1 μM). Cell lysates were analyzed by western blotting with an antibody directed against the C-terminal region of human MET, against PARP to assess caspase activation, or against GAPDH to assess loading. Arrowheads indicate full-length MET and the p40 MET caspase fragment. (**C**) MDCK cells were treated for 16 h with bafilomycin A1 (Baf; 5 nM) and/or Compound E (CpdE; 1 μM) or for 5 h with lactacystin (Lacta; 10 μM). Cell lysates were analyzed by western blotting with an antibody directed against the C-terminal region of human MET or ERK2. Full arrowheads indicate full-length MET and the MET intracellular domain (MET-ICD). Empty arrowheads indicate the MET C-terminal fragment (MET-CTF).

MDCK canine epithelial cells were stably transfected with a vector expressing the wild-type (WT) human MET receptor or a mutant variant thereof. We chose to express human MET variants in MDCK cells to efficiently detect transfected human construct and potentially generated fragments, since the canine MET receptor is not detected by the antibody directed against human MET. In addition, we previously demonstrated that the proteolytic cleavages of MET including those induced during apoptosis and necrosis and by PS-RIP occurred in these cells [14, 20]. The variants tested included juxtamembrane mutants found in lung cancer (R970C, P991S, or T992I) and a kinase domain mutant found in papillary renal cell carcinoma (M1250T) (Figure 1A). As expected, MDCK cells seeded at low density organized into small, compact islets. Expression of the M1250T variant, well known to trigger ligand-independent receptor activation, led to cell islet dissociation resembling the scattering triggered by HGF/SF (Supplementary Figure 1). In contrast, MDCK cells expressing WT MET or the R970C, P991S, or T992I variant did not display such scattering. This suggests that these MET variants are not constitutively activated in MDCK cells, as likewise shown previously in other cell types [29]. As shown in Figure 1B and 1C, the human MET receptors were expressed to comparable levels in the selected clones.

Since most of the previously described MET proteolysis occur within the juxtamembrane domain (Figure 1A), we evaluated whether juxtamembrane mutations modify them. First, MDCK clones expressing juxtamembrane mutants of MET were treated with staurosporin, an inducer of apoptosis. In all MDCK clones, treatment caused cleavage of PARP, a well-described caspase substrate (Figure 1B), confirming that caspase activity and apoptosis were triggered under these conditions. During apoptosis, as previously shown, MET was cleaved to generate a pro-apoptotic p40 MET^caspase^ fragment. Generation of p40 MET^caspase^ was observed with all the tested MET variants, demonstrating that juxtamembrane mutations do not affect caspase cleavage during apoptosis (Figure 1B). Similarly, during calcium stress-induced necrosis, MET juxtamembrane mutations did not alter generation of the p40 MET^calpain^ fragment (data not shown).

We next evaluated the effects of MET juxtamembrane mutations on the PS-RIP cleavages involved in receptor degradation under steady-state conditions [19]. As expected, treatment of MDCK cells with the lysosomal inhibitor bafilomycin or the γ-secretase inhibitor Compound E stabilized the MET C-terminal fragments (MET-CTF) detected near 55 kDa, generated by MET shedding. This stabilization was increased upon co-treatment with both inhibitors. Treatment with the proteasome inhibitor lactacystin stabilized as well the MET intracellular domain (MET-ICD, about 50 kDa) generated by gamma-secretase cleavage which was however barely detectable (Figure 1C). In MDCK cells expressing the juxtamembrane MET mutants, the tested treatments similarly stabilized the MET-CTF and MET-ICD fragments, demonstrating that these mutations do not affect cleavage by PS-RIP (Figure 1C). Taken together, these results show that the juxtamembrane mutations R970C, P991S, and T992I do not alter MET cleavages by caspases and calpains during apoptosis and necrosis respectively, nor PS-RIP cleavages under steady state condition.

### The R970C mutation promotes generation of a novel fragment, p45 MET

**Figure 2:**
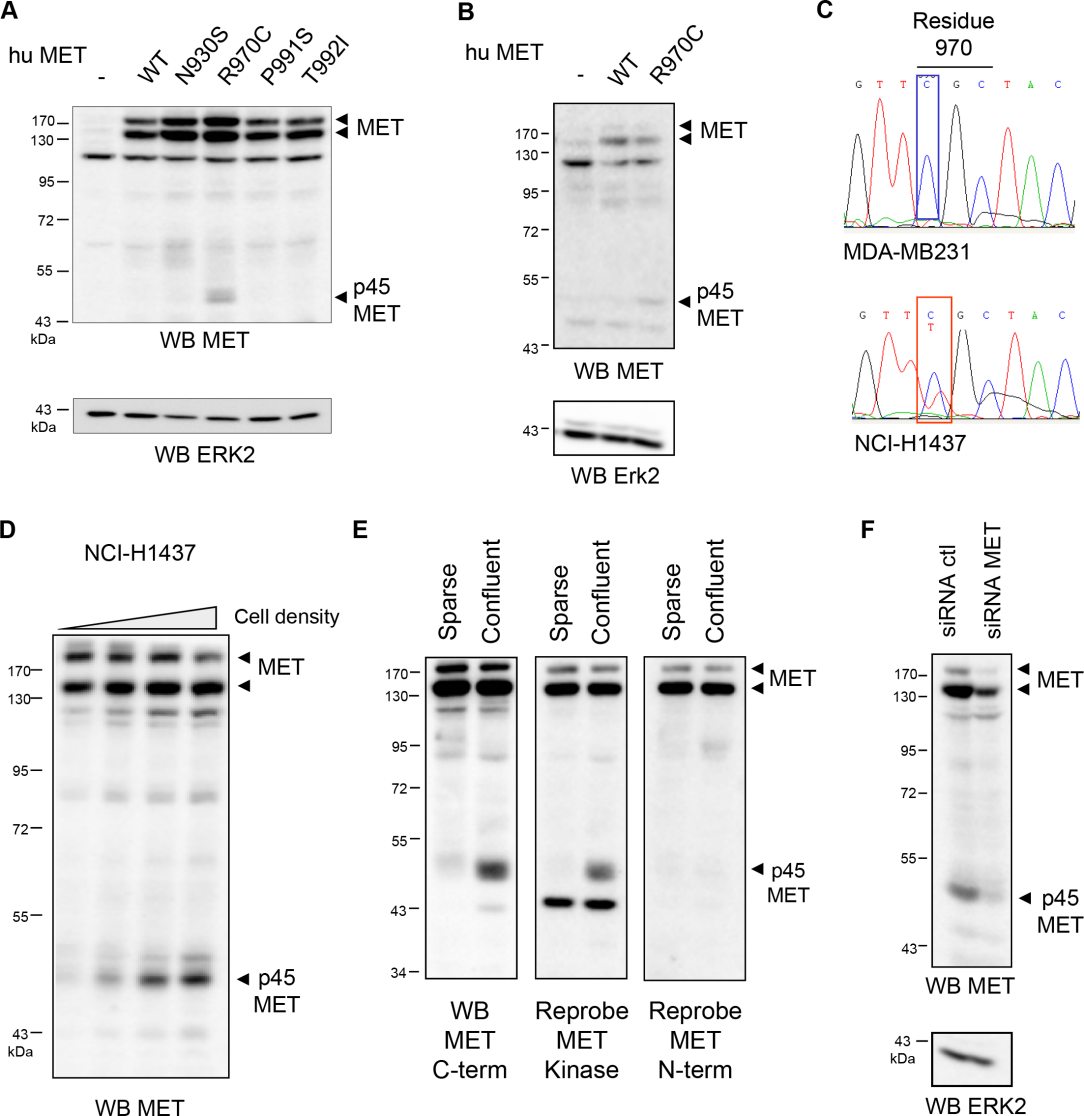
The R970C mutation promotes generation of a p45 MET fragment (**A**) Vectors expressing WT, N930S, P991S, T992I, or R970C human MET and (**B**) vectors expressing WT MET or R970C MET were used to transiently transfect MDCK or MCF-7 cells, respectively. (**C**) Genomic DNA of MDA-MB231 and NCI-H1437 cells was extracted and sequences corresponding to the MET juxtamembrane region were amplified by PCR and sequenced. (**D**) NCI-H1437 cells were seeded at 8, 4, 2, and 1 × 10^5^ cells/well in a 12-well plate and cultured for three days. (A, B, D) Cell lysates were analyzed by western blotting with an antibody directed against the C-terminal region of human MET and ERK2. (**E**) Sparse and confluent NCI-H1437 cells were cultured 48h. Cell lysates were analyzed by western blotting successively with an antibody directed against the C-terminal region of human MET (MET C-term), the kinase domain of MET (MET kinase) and the extracellular region of human MET (MET N-term). (**F**) NCI-H1437 cells were transfected with a control siRNA (ctl) or a pool of three siRNAs targeting MET. Cell lysates were analyzed by western blotting with an antibody directed against the C-terminal region of human MET and ERK2. Arrowheads indicate full-length MET and p45 MET.

In contrast to MDCK cells stably expressing WT human MET, we found that both MDCK and MCF-7 epithelial cells generate a novel fragment of about 45 kDa when transiently transfected with a vector expressing the R970C variant (Figure 2A and 2B). This fragment was not detected in cells overexpressing WT, P991S, or T992I MET or the N930S mutant, found in a melanoma cell line [33]. This suggests that the R970C mutation may promote heretofore undescribed proteolytic cleavages, generating the 45-kDa fragment, which we have named p45 MET.

To assess the involvement of this mutation in generating p45 MET, we evaluated MET expression in NCI-H1437, an NSCLC cell line carrying a monoallelic R970C mutation (Figure 2C). In NCI-H1437, in addition to the full-length MET, a 45-kDa fragment was easily detected. The level of this fragment was found to increase in cells cultured to high density (Figure 2D). The p45 MET fragment was recognized with two antibodies directed against the intracellular region of MET; while it was not with an antibody directed against the extracellular domain (Figure 2E). MET silencing in confluent NCI-H1437 cells was found to decrease both the MET and 45-kDa bands, indicating that p45 MET is indeed a MET fragment (Figure 2F).

**Figure 3:**
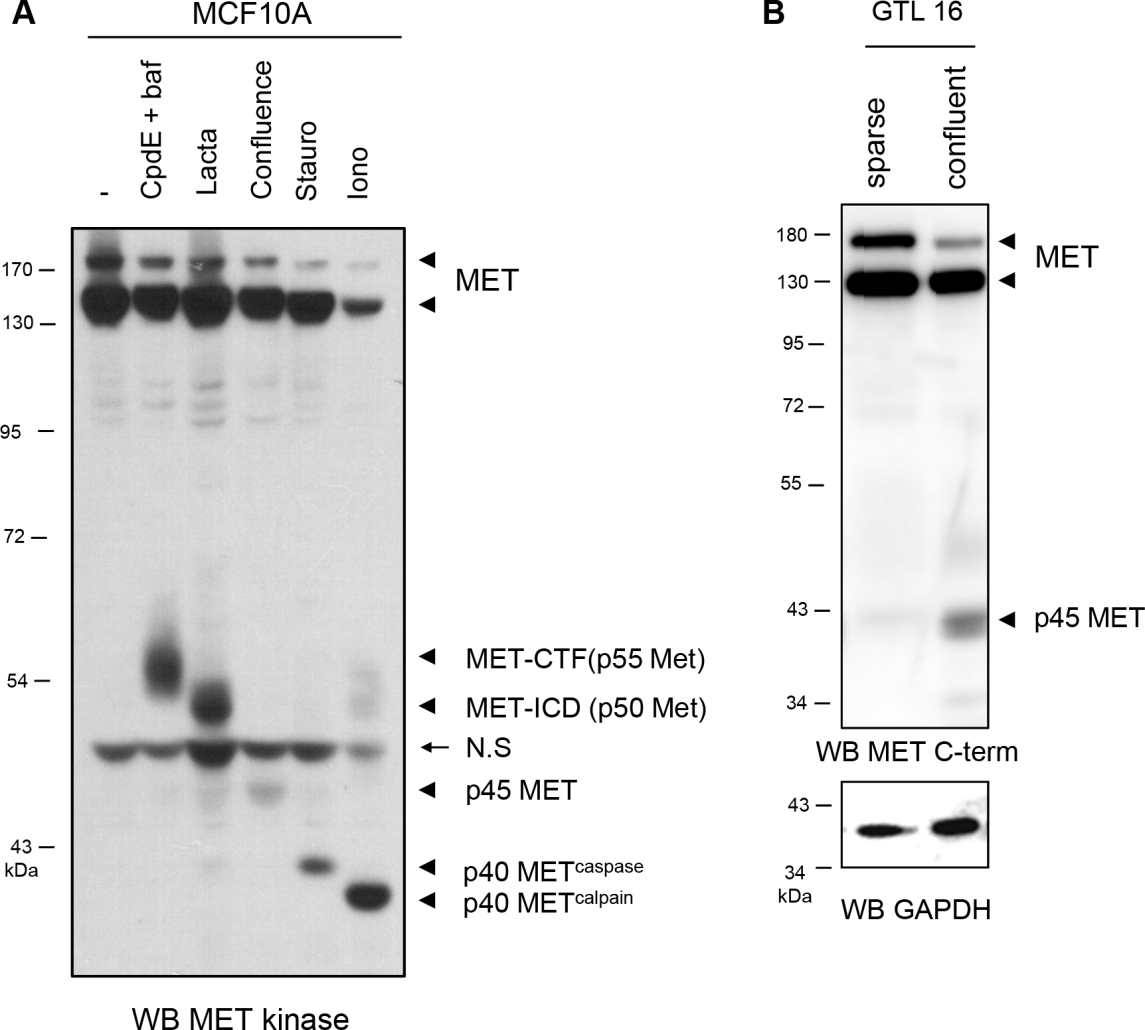
Generation of the MET fragments in epithelial cells (**A**) MCF10A cells were treated for 5 h with 1 μM compound E and 5 nM bafilomycin (CpdE+baf), for 5 h with 10 mM lactacystin (Lacta), for 6 h with 1 μM staurosporine (Stauro), for 1 h with 1 μM ionomycin (Iono), or cultured to high density (confluence). Cell lysates were analyzed by western blotting with an antibody directed against the kinase domain of MET. (**B**) Sparse and confluent GTL16 cells were cultured 48h. Cell lysates were analyzed by western blotting with an antibody directed against the C-terminal region of human MET (MET C-term) and GAPDH to assess the loading. Arrowheads indicate full-length MET, MET CTF (p55 MET), MET-ICD (p50 MET), p45 MET, the p40 MET^caspase^ and p40 MET^calpain^. Arrow indicates position of a non specific band (N.S).

In order to know if p45 MET fragment can be generated from WT receptor, mammary epithelial cell line MCF10A and gastric cancer cells GTL16 overexpressing MET were cultured in sparse and confluent conditions and MET expression was analyzed. P45 MET was detected in confluent MCF10A and GTL16, however to a lesser level than in NCI-H1437 (Figure 3A and 3B). To compare detection of all the known MET fragments, cell extracts of MCF10A cells containing generated or stabilized MET fragments were co-migrated. Labile fragments of 55 kDa (MET-CTF) and 50 kDa (MET-ICD) generated by PS-RIP were stabilized by lysosome and proteasome inhibitors respectively, p45 MET was generated in highly confluent cells and the fragments p40 MET^caspase^ and p40 MET^calpain^ were generated by inducing apoptosis or calcium stress-induced necrosis respectively (Figure 3A). All these fragments displayed different molecular weights confirming that MET undergoes multiple distinct proteolytic cleavages according to the culture conditions. It is worth noting that although p40 MET^caspase^ and p40 MET^calpain^ differ by only 30 amino acids, corresponding to about 3 kDa, this size difference was discernible in SDS-PAGE. Importantly, we recently showed that all these fragments, including this novel p45 MET fragment, were detected in lung tumor samples overexpressing MET [17]. Taken together, p45 MET fragment is generated in cell cultured under confluent condition and its generation is favored by the juxtamembrane R970C mutation.

### Calpain cleavage of MET is involved in generating p45 MET

**Figure 4:**
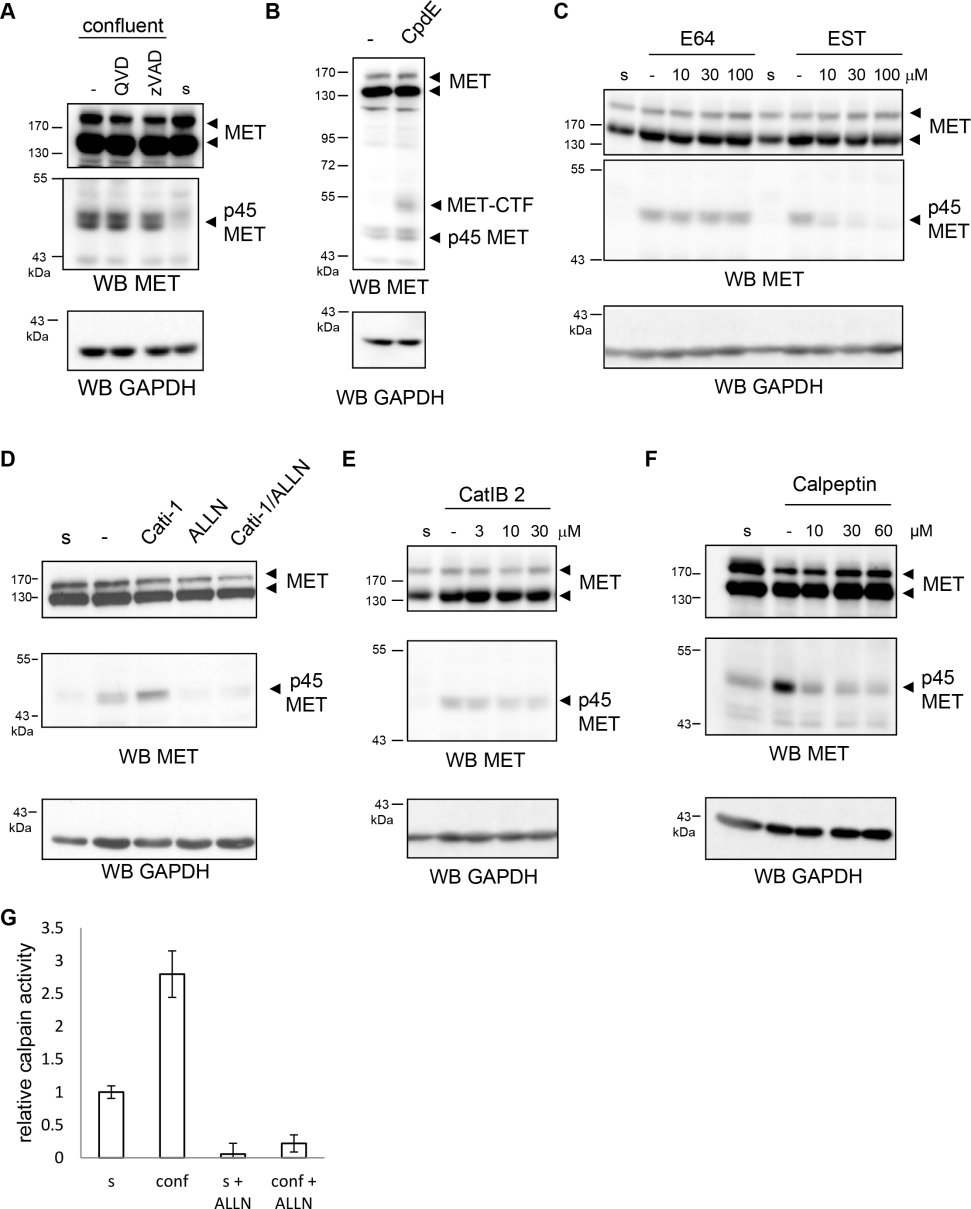
Involvement of proteases in p45 MET fragment generation Sparse (s) and confluent NCI-H1437 cells were treated 16h with various protease inhibitors: (**A**) with 20 μM pan-caspase inhibitor Q-VD and zVAD. (**B**) with 1 μM γ-secretase inhibitor Compound E; (**C**) 16 h with the indicated concentration of the non-permeating cysteine protease inhibitor E64 or the cysteine protease inhibitor EST; (**D**) with 20 μM cathepsin inhibitor Cati-1 or 20 μM calpain inhibitor ALLN; (**E**) with the indicated concentration of the cathepsin B inhibitor CatIB2; (**F**) with the indicated concentration of the calpain inhibitor Calpeptin. (A, B, C, D, E, F) Cell lysates were analyzed by western blotting with an antibody directed against the human MET C-terminal region and GAPDH. (**G**) Sparse (s) and confluent (conf) NCI-H1437 were lysed and total calpain activity was measured using Suc-LLVY-AMC synthetic substrate in presence or absence of 25 μM of calpain inhibitor ALLN (relative calpain activity to control; *n* = 3, ±standard deviation).

Detection of the p45 MET fragment favored by R970C mutation suggests novel proteolytic cleavage of MET. To test this hypothesis, we treated NCI-H1437 cells with various protease inhibitors. Pan-caspase inhibitors Q-VD and Z-VAD proved unable to inhibit p45 MET generation in confluent NCI-H1437 cells (Figure 4A). Similarly, generation of the fragment was unaffected by Compound E, a γ-secretase inhibitor, which as expected efficiently stabilized MET-CTF (Figure 4B). These results demonstrate that caspases and γ-secretase are not involved in p45 MET generation. Next, we treated NCI-H1437 cells with broad-spectrum inhibitors targeting main protease families. EST, which inhibits cysteine proteases, efficiently inhibited p45 MET generation in a dose-dependent manner, while its non-permeating homolog E64 did not (Figure 4C). In contrast, inhibitors of acidic or serine proteases did not inhibit fragment production (Supplementary Figure 2). Since calpains and cathepsins belong to the cysteine protease family, we tested their involvement in p45 MET generation. While cathepsin inhibitor Cati-1 and cathepsin B inhibitor catIB2 were unable to inhibit p45 MET formation (Figure 4D and 4E), the calpain inhibitor ALLN proved as effective as EST (Figure 4D). This result was confirmed with calpeptin, another calpain inhibitor (Figure 4F). Measure of calpain activity with fluorescent calpain substrate showed that, consistently with the increase in p45 MET production, calpain activity is about 3 times higher in confluent NCI-H1437 (Figure 4G). As expected, calpain inhibitor ALLN drastically decreased calpain activity. Taken together, these results indicate that although juxtamembrane MET mutations do not affect known proteolytic cleavages, the R970C MET variant undergoes cleavage by a protease of the calpain family.

### Ectopic expression of tagged p45 MET fragment increases epithelial cell scattering and motility

**Figure 5:**
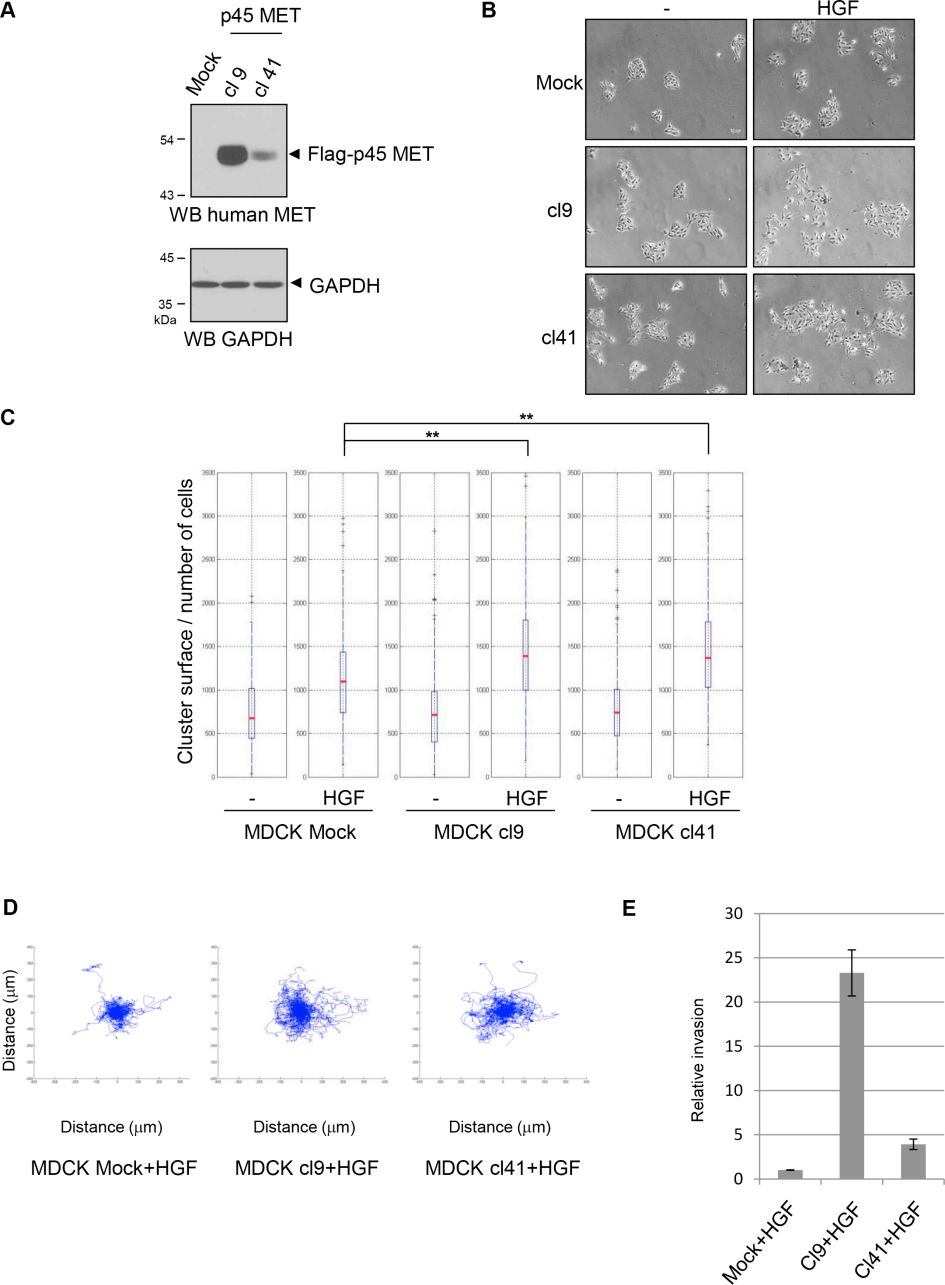
p45 MET fragment promotes cell migration of MDCK epithelial cells (**A**) Cell lysates from MDCK cells stably expressing (Clone 9 and 41) or not (mock) the flagged MET fragment beginning at the C970 amino acid (flag-p45 MET) were analyzed in duplicate by western blotting with an antibody directed against the human MET C-terminal region and GAPDH. Arrowheads indicate phosphorylated MET and the flag-p45 MET fragment. (**B**) MDCK stably expressing or not flag-p45 MET were seeded at low density. The next day the cells were treated 24h with 10ng/ml of HGF/SF. Cell islets were then fixed and stained (scale bar = 50 μm). (**C**) To evaluate cell scattering, cell density of more than 150 islets were calculated and shown as reciprocal values in a boxplot (the islet area divided by the number of cells in each islet). *p value* of student's *t* test is shown (**p < 0.001). (**D**) MDCK stably expressing or not flag-p45 MET were stained with fluorescent Dil-C12, seeded at low density and treated with HGF/SF 10ng/ml. Cells were tracked every 10min during 20 h by fluorescent video microscopy. Trajectories of 50 individual cells placed at the same origin were shown. (**E**) MDCK stably expressing or not flag-p45 MET were seeded at high density on modified Boyden chambers coated with Matrigel with 30 ng/ml of HGF in the bottom compartment. Two days later, cells of the transwell bottom surface were counted (relative number cells to control; *n* = 3, ±standard deviation).

**Figure 6:**
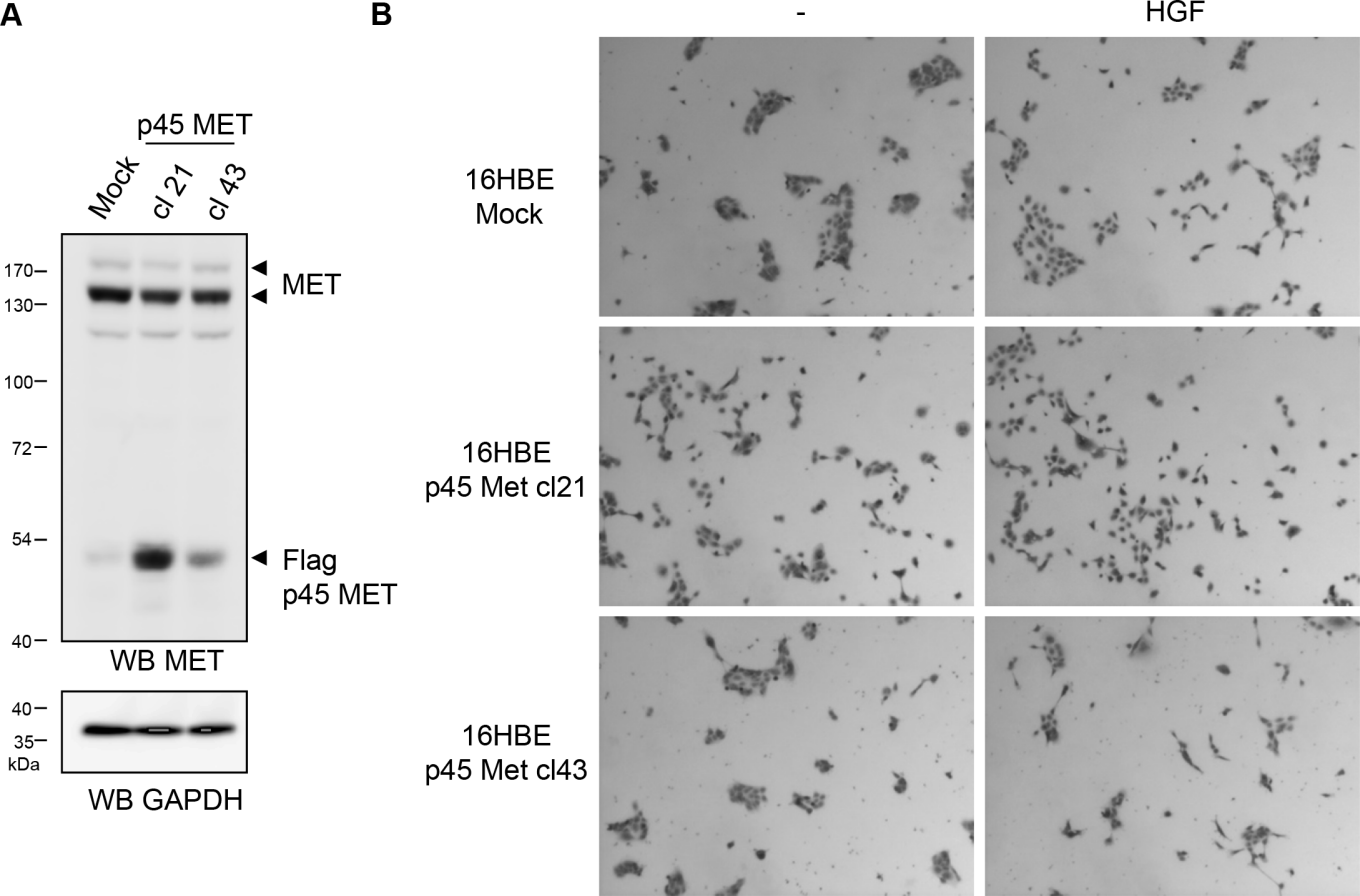
p45 MET fragment favors cell scattering of 16HBE epithelial cells (**A**) Cell lysates from 16HBE cells stably expressing or not the flagged MET fragment (flag-p45 MET) were analyzed by western blotting with an antibody directed against the flag tag and GAPDH. Arrowheads indicate the p45 MET fragment and GAPDH. (**B**) 16HBE stably expressing or not flag-p45 MET were seeded at low density. The next day the cells were treated 24 h with 10 ng/ml of HGF/SF. Cell islets were then fixed and stained.

In order to evaluate potential role of p45 MET fragment in MET dependent biological responses, we stably expressed in the MDCK epithelial cells, a flag tagged p45 MET fragment from the amino acid C970 to the C-terminal end. We selected two clones expressing flag-p45 MET fragment by western blotting with an anti-human MET antibody at an apparent molecular mass close to the p45 MET generated from MET R970C mutant (Figure 5A). Similar detection was obtained with an anti-flag antibody (data not shown). In the MDCK clones, expression of p45 MET did not modify actin stress fibers organization visualized through phalloidin staining nor their proliferation (Supplementary Figure 3A and 3B). However, clones expressing flag-p45 MET displayed increased cell scattering in response to HGF/SF compared to wild type MDCK cells (Figure 5B and 5C). Trajectories of fifty individual cells tracked by fluorescent video microscopy showed that the clones expressing flag-p45 MET displayed a higher motility in response to HGF/SF treatment (Figure 5D). Velocity of cells measured in mm/h confirmed this higher motility (Supplementary Figure 3C). Furthermore, measure of invasion through modified Boyden chamber coated with Matrigel showed that ligand stimulated MDCK cells expressing p45 MET seeded at high density were more invasive compared with the mock cells. The stronger response was observed with the clone 9 expressing the higher level of fragment, suggesting that intensity of the invasion response is associated with the expression level of p45 MET (Figure 5E). No invasion was observed in all MDCK cells unstimulated by HGF/SF. As expected, in these MDCK cells, HGF/SF stimulation induced efficient full length MET tyrosine phosphorylation, while it induced very low Flag-p45 MET phosphorylation barely detectable at 10 and 30 minutes (Supplementary Figure 4). Similarly, in NCI-H1437 cells, endogenous p45 MET was not found phosphorylated with or without HGF stimulation (Supplementary Figure 5A). In contrast, in sparse and confluent conditions, full length MET was found phosphorylated without HGF, while ligand stimulation induced weak or undetectable increase of the receptor phosphorylation (Supplementary Figure 5A and S5B). Nevertheless, HGF/SF treatment increased ERK phosphorylation both in sparse and confluent conditions, demonstrating effective activation of this downstream signaling pathway in the NCI-H1437 cells. As expected, the MET tyrosine kinase inhibitor PHA-665752 inhibited both MET and ERK phosphorylation induced or not by HGF (Supplementary Figure 5B). It is worth noticing that PHA-665752 treatment increased full length MET expression, a likely consequence of the lysosomal dependent degradation of the activated receptor. To assess increase of epithelial cell motility by p45 MET, we stably expressed the fragment in the human bronchial epithelial cell line 16HBE. We selected two clones expressing the fragment (Figure 6A). The clones expressing flag-p45 MET displayed increased cell scattering in response to HGF/SF compared to wild type 16HBE and the clone expressing the higher level of p45 MET display even basal scattering without HGF/SF stimulation (Figure 6B). Taken together, these results show that p45 MET fragment favors epithelial cells scattering, motility and invasion induced by HGF/SF.

## DISCUSSION

MET mutations are found in 3–10% of lung cancers and mostly affect the juxtamembrane domain. Because the amino acid substitutions within the juxtamembrane domain of MET do not cause kinase activation, it has been proposed that they could be polymorphisms [29]. Nevertheless, other studies have demonstrated that the juxtamembrane MET mutations R970C, P991S, and T992I favor migration and colony formation in soft agar and increase levels of reactive oxygen species, a phenomenon which may participate in cell transformation [28, 34]. In addition, the mouse strain SWRJ, carrying the R968C MET variant (corresponding to the human R970C variant), displays susceptibility to lung tumor development when exposed to a carcinogen [32]. Since it is unknown how the R970C mutation affects MET regulation, it may be important to characterize the molecular mechanisms that are modified because of these variants to further understand their involvement in lung tumorigenesis.

We show here that MDCK epithelial cells expressing wild-type, R970C, P991S, or T992I MET do not display constitutive scattering, in contrast to cells expressing the activated-kinase mutant MET M1250T. This confirms that the juxtamembrane mutations do not lead to constitutive activation of the receptor. The absence of constitutive MET activation by the juxtamembrane mutations suggests that other mechanisms that do not directly affect kinase activity could be involved in their deregulation.

We previously demonstrated that the proteolytic cleavages of MET are involved in inactivating the receptor by separating the ligand-binding region from the tyrosine kinase domain. Inhibition of these cleavages could thus favor MET stability and hence MET activation. In ovarian carcinoma, a MET mutation has been found in the C-terminal caspase site (DEVD_1380_N) [35]. This suggests that inhibition of MET caspase cleavages could be involved in MET deregulation in cancers. Although MET juxtamembrane mutations in lung tumors do not target the juxtamembrane caspase site or the predicted γ-secretase cleavage site often located in the last amino acids of the transmembrane domain, we hypothesized that these mutations might modify the structure of the domain, thus altering the proteolytic cleavages. We demonstrate here, however, that the juxtamembrane mutations R970C, P991S, and T992I do not abolish MET cleavages by caspases during apoptosis, by calpains during necrosis or by secretases under steady-state conditions.

Interestingly, we find that one of the MET variants, the R970C mutant, favors generation of an as yet undescribed 45-kDa fragment. We initially observed this fragment upon overexpression of MET in transiently transfected MDCK cells. However, it is worth noticing that in MDCK cells stably transfected with the same MET variant, we did not observe generation of the fragment even when cells are cultured to high density. This could be a consequence of the strong difference in MET expression level in transiently transfected cells compare to the stable cell lines. Thus, the massive expression of the MET R970C in the transiently transfected cells could favor detection of the modest amount of fragment generated in MDCK cells. Alternatively, the massive expression of the mutated MET could favor the calpain dependent cleavage. In any event, in transiently transfected cells including the MDCK and MCF7 cells, p45 MET was generated only from the R970C mutant, while it was not produced from the WT or the other mutated MET receptors. Generation of p45 MET was also found in lung cancer cell lines NCI-H1437 expressing mutated MET R970C. Generation of the fragment was inhibited by broad-spectrum cysteine protease inhibitors and also by the calpain inhibitors ALLN and calpeptin. This suggests that calpain cysteine proteases are involved in this processing. Inhibition of other cysteine proteases such as caspases and cathepsins did not prevent p45 MET generation. The apparent molecular size of the p45 MET fragment is consistent with a juxtamembrane cleavage occurring near position 970, but it is difficult to predict the exact cleavage site, since consensus amino-acid sequences recognized by calpains have not been described. Currently, the spatial conformation of a substrate is thought to be a more important factor for calpain cleavage, even though the exact mechanism is still unknown [36]. Interestingly, tyrosine kinase receptors have recently been predicted to be calpain substrates [37]. We also find that cell density increases generation of the p45 MET fragment. Consistently, confluent NCI-H1437 cells display higher calpain activity compare to cells cultured in sparse condition. Such results have already been described for calpain cleavage of Frizzled-7, a G protein coupled receptor involved in cell migration. This cleavage is suggested to be a downregulation mechanism of the membrane pool of Frizzled-7, notably at high cell density, allowing correct turnover of this protein crucial for cell motility [38]. In epithelial cells expressing wild type MET, the p45 MET fragment is not detected when cells are cultured to low density. In contrast in the same cells cultured to high density, the fragment becomes detectable, however at lesser extent that the NCI-H1437 cells expressing mutated MET R970C. Thus, we propose that calpain cleavage of MET can occur on wild type receptor, but it is favored by the R970C variant.

Calpains are a family of cytoplasmic calcium-activated proteases involved in many cellular events. They can promote cell death through cleavage of components of the apoptotic machinery, such as caspases and Bcl-2 family proteins, or, in the context of an altered apoptotic pathway or of strong calcium stress, induce necrosis-like caspase-independent cell death [36, 39, 40]. Beside their involvement in cell death, calpains can promote cell migration through cleavage of focal adhesion proteins allowing their turnover [41, 42]. According with the involvement of calpains in various biological responses, we recently demonstrated that during calcium stress-induced necrosis, MET is cleaved by calpain 1 and 2 to generate an about 40 kDa fragment. This fragment is quite similar to p40 MET^caspase^ during apoptosis, but it is unable to promote cell death [17]. We demonstrated here that calpains are also involved in generation of a novel p45 MET. Thus, calpains would be able to cleave MET in two distinct cellular conditions, (i) a cleavage occurring at T1036 during necrosis to generate p40 MET^calpain^, (ii) a cleavage at or near the position 970 favored by cell density and R970C mutation to generate p45 MET. It would be interesting to further characterize the proteases involved, since distinct calpains could be responsible for these two proteolytic processes.

In MET R970C, calpain cleavage within the juxtamembrane domain separates the extracellular ligand-binding region from the intracellular kinase domain. The MET juxtamembrane cleavages during apoptosis and necrosis and by gamma-secretase likewise separate these functional domains, preventing ligand activation of the receptor [43]. In addition, during apoptosis and necrosis, the cleavages are accompanied by decrease of full length MET, demonstrating its degradation during these processes [17]. In contrast, in confluent NCI-H1437 cells, generating high level of p45 MET, expression of full length MET does not decrease. This suggests that cleavage generating p45 MET is not involved in the full length receptor degradation.

We have also previously demonstrated, however, that some intracellular fragments of MET can be active: the p40 MET^caspase^ favors cell death through mitochondrial permeabilization, a crucial step in the apoptotic process [16]. To evaluate the involvement of p45 MET in biological responses induced by full length MET, we ectopically expressed flag-p45 MET fragment in MDCK and 16HBE epithelial cells. We reconstitute the fragment from the mutated cystein residue 970 to the C-terminal end. The apparent molecular mass of the reconstituted fragment is close to endogenous p45 MET confirming that the calpain proteolytic cleavage of MET R970C occurs at or near the mutation. Interestingly, we found that ectopic expression of p45 MET in MDCK cells increases cell scattering, motility and invasion in response to HGF/SF. Similar increase of cell scattering was observed in human bronchial epithelial cell line 16HBE. However, p45 MET expression in MDCK cells did not induce morphological changes or modification of proliferation rate without HGF/SF stimulation. Full length MET transduces intracellular signaling through its tyrosine phosphorylation notably involved in recruitment of intracellular adaptors. Nevertheless, endogenous p45 MET does not display tyrosine phosphorylation, even following HGF/SF treatment and stably expressed flag-p45 MET fragments displayed only very weak phosphorylation under HGF/SF stimulation, suggesting that it does not promote epithelial cell scattering through the same mechanisms than the phosphorylated full length MET able to induce activation of canonical signaling pathways.

Interestingly, many fragments of membrane receptors, including receptor tyrosine kinases, can regulate transcriptional responses upon their translocation into the nucleus [44]. For instance, ERBB4 intracellular fragment (ERBB4-ICD), generated by its proteolytic processing by TACE and gamma-secretase upon its ligand stimulation, is able to associate with the transcriptional regulator YAP [45]. Furthermore, it has been recently shown that ERBB4-ICD/YAP transcriptional response is involved in migration of mammary epithelial cells [46]. In the same line, an intracellular C-terminal fragment of MET fused to the Gal-4 DNA binding domain has been shown to display transactivation properties, which was inhibited by YAP silencing [47]. This suggests that a MET fragment might act as a transcription regulator involving the transcriptional regulator YAP. Because p45 MET plays an active role on HGF/SF induced scattering and invasion, evaluation of its transcriptional potential might be an interesting issue.

Besides proteolytic cleavages, the juxtamembrane region of MET is targeted by other negative regulatory mechanisms, involving ligand-dependent phosphorylation. First, in response to HGF/SF, phosphorylation of MET at tyrosine residue 1003 leads to recruitment of the ubiquitin ligase CBL, causing ubiquitination and lysosomal degradation of the receptor [48]. Second, ligand stimulation induces phosphorylation of serine residue 985, involved in downregulating MET kinase activity [49]. Because the P991S and T992I mutations are located near these negative regulatory sites, it would be interesting to evaluate whether they might affect juxtamembrane phosphorylation in response to the ligand.

Two recent studies have reported that R970C mutant is present in blood samples from healthy people [29] and in samples from normal lung [31], suggesting that R970C mutant could be a polymorphism. However, the low frequency of this variant in healthy people as well as in tumors samples (about 1% in each case) did not allow strong conclusion. Nevertheless, some polymorphisms initially found in tumor samples are susceptibility factors able to promote tumorigenesis. Several arguments are in favor of R970C variant as a susceptibility factor in lung cancer. First, the SWR/J mouse strain, expressing the R968C MET variant, displays susceptibility to lung tumor development when exposed to a carcinogen [32]. Second, it has been recently shown that ALK-rearranged pulmonary adenocarcinomas display higher frequency of R970C variant (about to 4% compared to 1% in healthy persons), suggesting association between this MET mutation and ALK rearrangement [31]. Finally, we demonstrate here that ectopic expression of p45 MET fragment can favor epithelial cells scattering, motility and invasion induced by HGF/SF in a tyrosine kinase activity-independent manner. Taken together, increase of calpain cleavage of MET by R970C mutation demonstrates that besides the well-described deregulation of MET kinase activity, unexpected deregulation of other mechanisms could target the MET receptor, enabling it to participate in cell transformation. It is worth noticing that we recently demonstrated that the p45 MET, like the other characterized MET fragments, is present in lung tumors overexpressing MET [17], suggesting that this fragment could play active role during tumorigenesis.

## MATERIALS AND METHODS

### Cytokines, drugs, and cell cultures

The caspase inhibitors QVD-FMK and zVAD-FMK, the H+-pump inhibitor bafilomycin A1, the calpain inhibitors ALLN, the calpeptin and the cathepsin inhibitors Cati-1 and CatIB 2 were purchased from Calbiochem (San Diego, CA, USA). Staurosporin and the proteasome inhibitor lactacystin were purchased from Sigma (St Louis, MO, USA). The γ-secretase inhibitor Compound E was purchased from Alexis/Coger (Lausen, Switzerland). The aspartyl protease inhibitor pepstatin, the trypsin serine protease inhibitor TLCK, the chymotrypsin serine protease inhibitor TPCK, and the cysteine protease inhibitors E64 and EST were purchased from Calbiochem. Human recombinant HGF/SF was purchased from Peprotech (Rocky Hill, NJ, USA). The MET kinase inhibitor PHA-665752 was purchased from Promega (Madison, WI, USA).

Madin-Darby canine kidney (MDCK) epithelial cells, GTL16 cells and MDA-MB-231 cells were cultured in Dulbecco's Modified Eagle's Medium (Invitrogen, Carlsbad, CA, USA) supplemented with 10% fetal bovine serum (FBS, Invitrogen) and 1% antibiotics (10000 U/ml penicillin - 10000 μg/ml streptomycin, Invitrogen). NCI-H1437 and MCF-7 were cultured in RPMI 1640 (Gibco) supplemented with 10% FCS and antibiotics. Human bronchial epithelial cell line 16HBE14o was grown in MEM-GlutaMAX, 10% FBS and antibiotics. Cells were cultured at 37°C in a water-saturated 5% CO_2_ atmosphere.

### Antibodies

Mouse monoclonal antibody directed against human MET (#3148) and rabbit polyclonal antibody directed against the phosphorylated tyrosine (Y 1234/1235) of MET (#3126) were purchased from Cell Signaling Technology (Danvers, MA, USA). Mouse monoclonal antibody against the kinase domain of MET (3D4) was purchased from Invitrogen. Mouse monoclonal antibody directed against the N-terminal domain of MET (DL-21) was kindly provided by Dr Sylvia Giordano (University of Torino Medical School, Italy) [50]. Mouse monoclonal antibody against lamin B2 (33–2100) was purchased from Zymed. Mouse monoclonal antibody against GAPDH (6C5-32233), rabbit polyclonal antibody against the C-terminal domain of human PARP-1 (H250), and antibody against ERK2 (C14) were purchased from Santa Cruz Biotechnology (Santa Cruz, CA, USA). Green-fluorescent Alexa fluor 488 conjugated anti-mouse IgG (H+L) and phalloidin– Alexa Fluor 546 were purchased from Invitrogen. Rabbit polyclonal antibodies against the Flag epitopes were purchased from Sigma (St Louis, MO, USA).

### Plasmid constructs

The vector expressing full-length human MET was constructed as follows. The sequences encoding WT human MET and the kinase-dead MET K1108A variant, previously described [13], were cut out of PRS2 hu MET (kindly provided by Dr G. Vande Woude, Van Andel Research Institute, MI, USA) by EcoRI restriction and cloned into the pCAGGS vector (kindly provided by Dr P. Mehlen, Centre Léon Bérard, Lyon, France).

Expression vectors for human WT MET and for the N930S, R970C, P991S, and T992I MET variants were constructed as follows. Mutations were created with the QuickChange site-directed mutagenesis system of Stratagene, using pRS2-human MET as a template and the following primers:

N930S: 5′-GTTCAACCAGATCAGAGTTTCACAGGATTG and

3′-CAATCCTGTGAAACTCTGATCTGGTTGAAC

R970C: 5′-GCAGTGAATTAGTTTGCTACGATGCAAGAG and

3′-CTCTTGCATCGTAGCAAACTAATTCACTGC

P991S: 5′-GCCCGAAGTGTAAGCTCAACTACAGAAATG and

3′-CATTTCTGTAGTTGAGCTTACACTTCGGGC

T992I: 5′-CGAAGTGTAAGCCCAATTACAGAAATGGTTTC and

3′-GAAACCATTTCTGTAATTGGGCTTACACTTCG

The mutated pRS2-MET plasmids were digested with Age-1/Cla and the resulting fragments were subcloned in pCAGGS-MET expression vectors.

Flag-p45 MET was constructed as follows. The human MET fragment was amplified by PCR with the following primers 5′-ATGGATCCTGCTACGATGCAAGAGTACACAC -3′ containing a BamH1 restriction site and 5′- CGCGCTCGAGCTATGATGTCTCCCAGAA-3′ containing a Xho1 restriction site. The PCR product was subcloned into pcDNA3-Flag between the BamH1 and Xho1 restriction sites.

### Transfections

Transfections of MCF7 and MDCK cells with the reagents polyethyleneamine (PEI) Exgen 500 (Euromedex), and Lipofectamine (Invitrogen) were performed as previously described [13]. For stable transfections of MDCK cells, two days after transfection, cells were split into four 100-mm dishes containing DMEM-10% FCS, and the next day the medium was supplemented with 800 μg/ml G418 (Gibco). Resistant clones were isolated after 10 days and clones expressing the transfected constructs were selected by western blotting.

### RNA interference

For MET silencing, NCI-H1437 cells were harvested. 5.10^5^ cells were incubated with 3 μL Lipofectamine 2000 (Invitrogen) and mixed with a pool of three stealth siRNAs (30nM each) (Invitrogen) targeting MET [5′-CCAUUUCAACUGAGUUUGCUGUUAA-3′, 5′-UCC AGAAGAUCAGUUUCCUAAUUCA-3′, 5′-CCGAGGG AAUCAUCAUGAAAGAUUU-3′]. Four transfections of 5.10^5^ cells were pooled to plate 2.10^6^ cells in a 6-well plate in complete medium.

### Western blotting

Western blotting was performed as previously described [51]. All samples were resolved in 10% SDS-polyacrylamide gel electrophoresis. For quantification of protein expression, luminescence was captured with a digital imaging using a cooled charge coupled device (CCD) camera (LAS 3000, Fuji) and quantification was performed using Multigauge V3.0 software. The background adjusted volume was normalized to empty well.

### Immunofluorescence staining

Cells were grown on glass coverslips for 24 h, washed, fixed and permeabilized 10min in methanol:acetone (1:1) at –20°C. The cells were washed and blocked 30 min in PBS 0.2% casein. The cells were washed and the nuclei counterstained with Hoechst 33258. For F-actin staining, cells were incubated with phallodin-Alexa Fluor 546. Coverslips were mounted with Glycergel mounting medium (Dako, CA). Slides were observed in oil immersion with an AxioImager Z1 (Carl Zeiss), numerical aperture: EC-Plan Neofluar 40x NA 1.3, with an AxioCam mRm camera and Zen Blue acquisition software.

### Scattering, motility, invasion and proliferation analysis

Microscopy was performed on a Zeiss AxioObserverZ1 fluorescence microscope equipped with a 5x Fluar lens (numerical aperture [NA]0.25), an Axio mRm camera, a Lambda DG4 excitation source (Sutter Instrument Company), a DsRed Filter (Zeiss Filter Set 43HE) and an incubation Unit (XL Unit, Pecon). For time-lapse microscopy, the interior of the incubator surrounding the entire microscope system was heated to 37°C and the CO_2_ level was regulated to 5%. 800 cells were labeled with DilC12 (15μg/ml; Becton Dickinson) and seeded in Ibidi 15 μ slide 8 wells (GmbH, München, Germany). Images were acquired with the Zen software (Zeiss) at a rate of 1 image per time interval of 10min. The Plugin MTrack2 implemented in the Fiji software (NIH) enabled to obtain the trajectory of each cell. Data were processed with Matlab (Mathworks) to calculate the mean displacement for each cell. For scattering, 2500 cells/well were seeded onto 12-well plates in DMEM-10% FCS. The next day, they were treated or not 24h with 10ng/ml HGF/SF in DMEM-0.5% FCS. Cells were stained with Carazzi's hematoxylin and eosin. Bright-field images were acquired in mosaic mode, thresholded by the Fiji software (NIH) and cells were localized by a maxima detection algorithm. The next steps of analysis were done with Matlab. After a k-means clustering to individualize clusters, a convex hull detection function enabled to detect the outer border of each cluster and to measure its area. For proliferation, 5000 cells/well were seeded onto 24-well plates in RPMI medium with 10% FCS. The number of migrated cells was then counted. Cell proliferation was assessed in triplicate during 4 days after seeding. Cells were counted with Beckman Coulter Z2. Invasion was assayed in Transwell cell culture chambers with a polycarbonate filter-membrane of 8 mm-pore size in 24-well plates coated with Matrigel (BD Biosciences). MDCK cells (100 000) were seeded to the upper compartment of the chamber in serum-free medium. The lower chamber was filled with serum-free medium containing 30 ng/ml of HGF. After 48 h of incubation at 37°C, the non-migrating cells were removed by wiping with a cotton swab. The cells on the lower surface of the membrane were fixed with PFA and stained with Hoechst-33342.

### Calpain activity

Cells were lysed in Buffer A (HEPES 10 mM pH = 7.4, 10 mM KCl, 1.5 mM MgCl2, 0.5%NP-40). Lysates were clarified by centrifugation at 4°C, and protein concentration was determined with Pierce BCA Protein Assay kit (Thermo Scientific, Massachusetts USA). Proteins were diluted at the same concentration in Buffer A and 50 μl of each sample were transferred to a 96-well plate. 50 μl of calpain substrate Suc-LLVY-AMC (Anaspec, CA USA) diluted at 1:100 in Buffer A were added to each well and fluorescence (Ex/Em = 354 nm/442 nm) was monitored every 5min for 1h at room temperature with a fluorimeter (BMG Labtech FLUOstar OPTIMA). The slopes of the activity curves were calculated and expressed with respect to the control condition.

## SUPPLEMENTARY MATERIALS FIGURES AND TABLES


