# Supplementary material for: MET receptor variant R970C favors calpain-dependent generation of a fragment promoting epithelial cell scattering
Source: Oncotarget. 2017 Jan 4;8(7):11268–83. doi: 10.18632/oncotarget.14499 (PMC5355264; doi:10.18632/oncotarget.14499)
Supplement: Supplementary file 1 [file oncotarget-08-11268-s001.pdf]

MET receptor variant R970C favors calpain-dependent generation of a fragment promoting epithelial cell scattering

Supplementary Materials

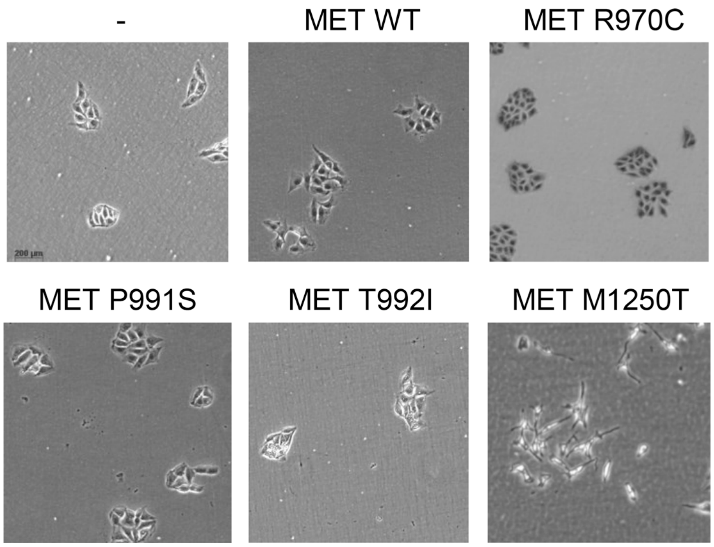

Supplementary Figure 1: MDCK cells stably expressing wild-type human MET (MET WT) or a variant mutated in the juxtamembrane domain (R970C, P991S, T992I) or kinase domain (M1250T) were seeded at low density. The next day cell islets were fixed and stained.

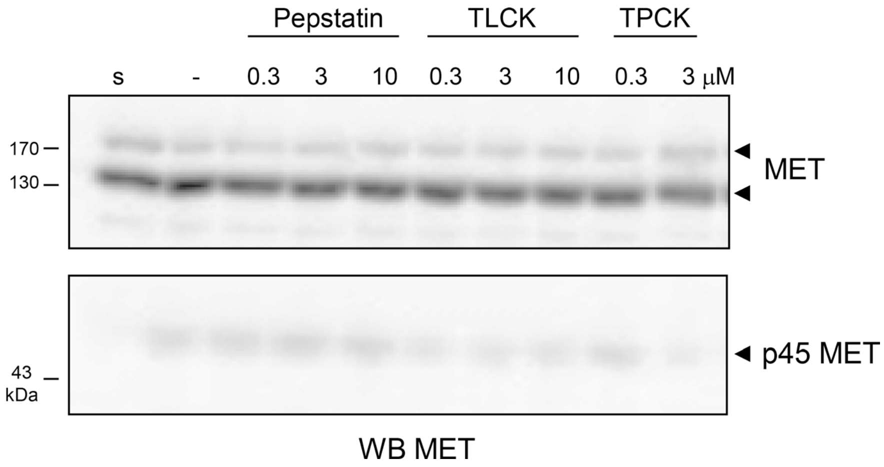

Supplementary Figure 2: Sparse (s) and confluent NCI-H1437 cells were treated for 16 h with the indicated concentration of the aspartyl protease inhibitor pepstatin, the trypsin serine protease inhibitor TLCK, or the chymotrypsin serine protease inhibitor TPCK. Cell lysates were analyzed by western blotting with an antibody directed against the C-terminal region of human MET. Arrowheads indicate full-length MET and the p45 MET fragment.

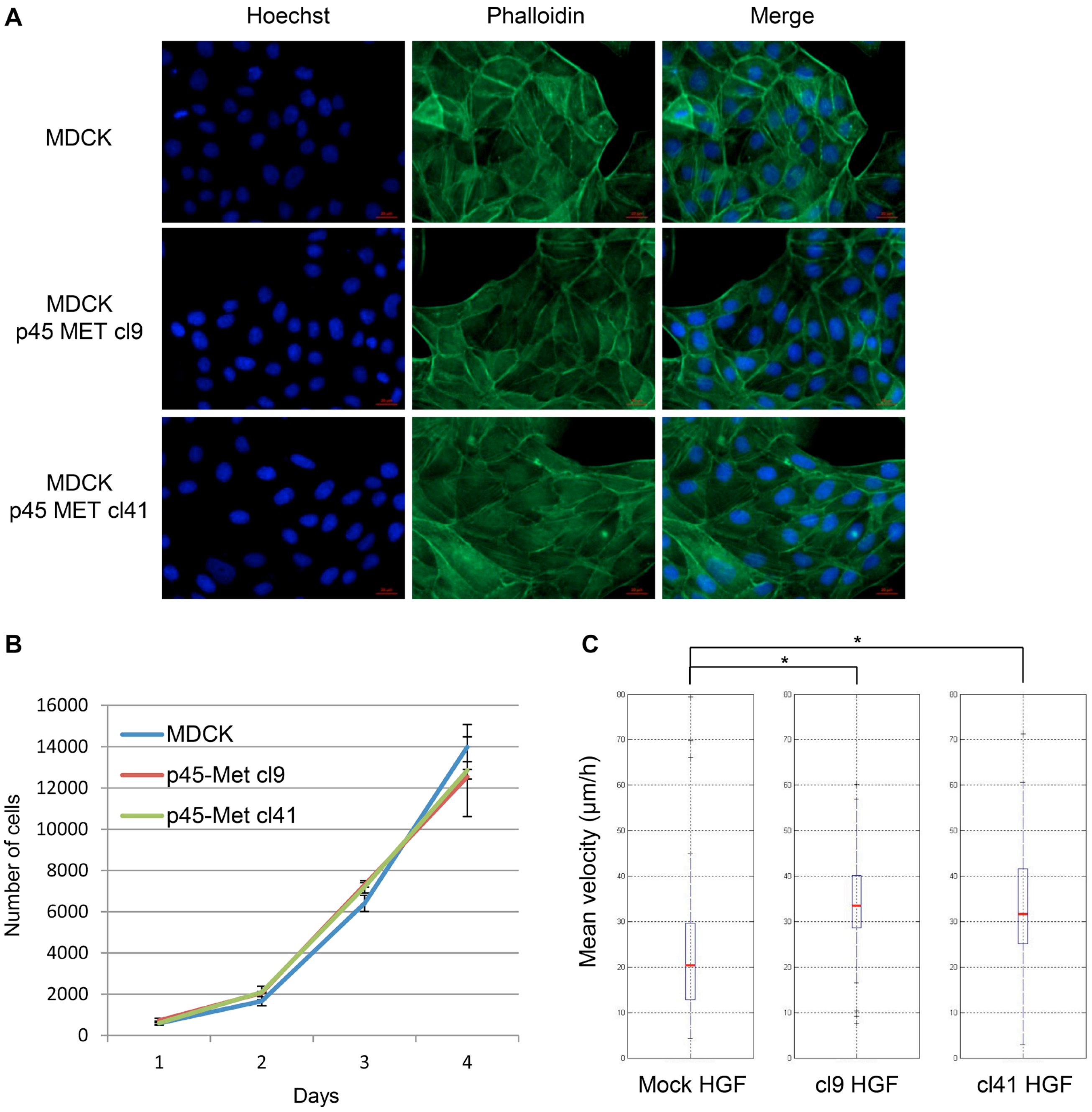

Supplementary Figure 3: (**A**) MDCK cells expressing flagged p45 MET (MDCK p45 MET cl9 and cl41) were fixed and stained for F-actin with phalloidin (green staining) and their nucleus stained with Hoechst (blue staining). An overlay of the two stains is shown (merge) (scale bar = 20 µm). (**B**) MDCK cells expressing or not flagged p45 MET (MDCK p45 MET cl9 and cl41) were seeded at the same density. Each day, cells were counted ($n = 3$; –/+ SD). (**C**) MDCK cells stably expressing or not flag-p45 MET were stained with fluorescent Dil-C12, seeded at low density and treated with HGF/SF 10 ng/ml. Trajectory of 50 individual cells was tracked every 10 min during 20 h by fluorescent video microscopy. Velocity of cells in µm/h was shown in a boxplot. p value of student's t test is shown ($^*p < 0.005$).

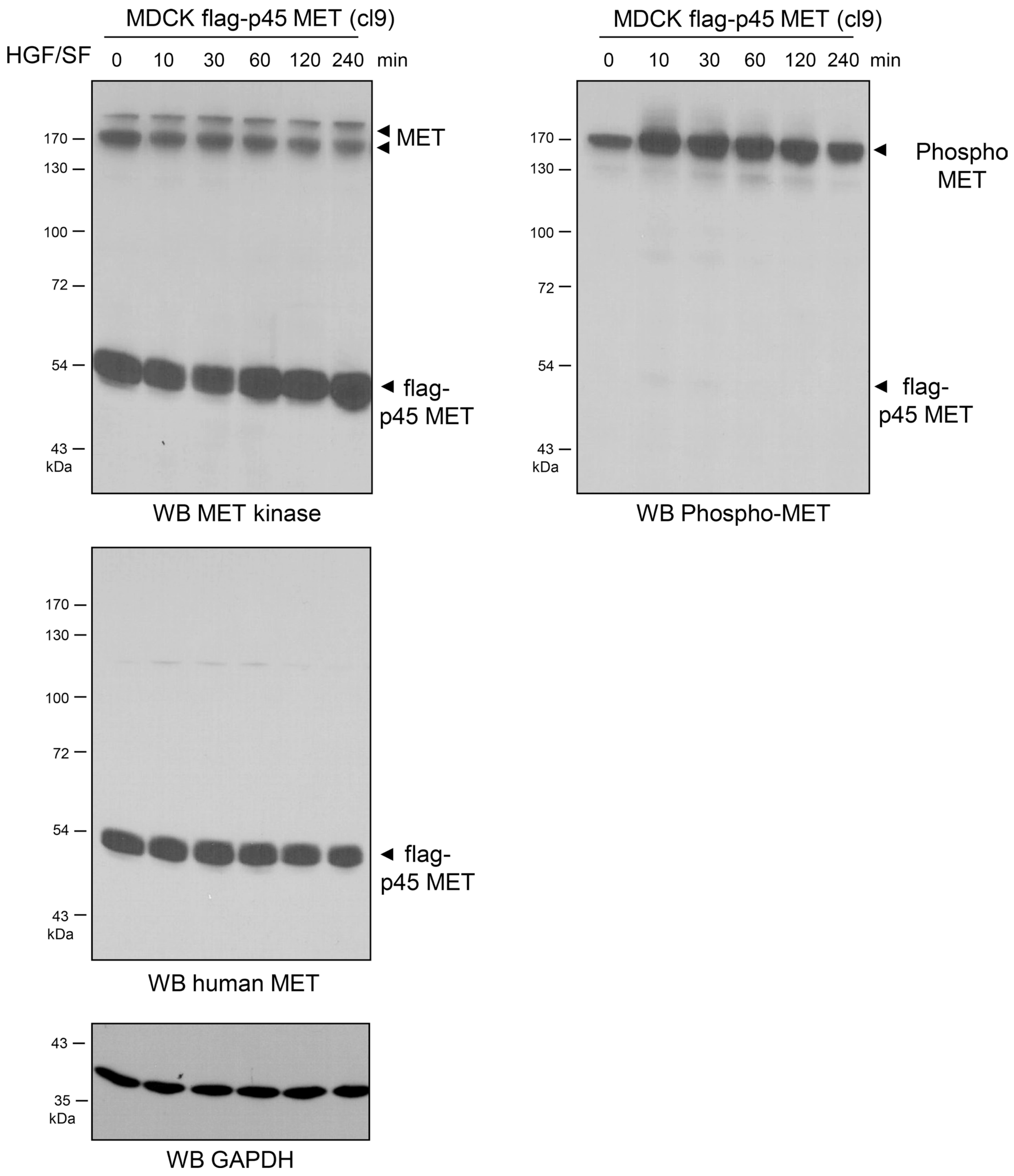

Supplementary Figure 4: MDCK cells expressing flagged p45 MET (MDCK flag-p45 Met clone 9) were treated with 30 ng/ml of HGF/SF during the indicated time. Cell lysates were analyzed by western blotting with an antibody directed against the kinase domain of MET recognizing endogenous canine MET and transfected human p45 MET. Membrane was successively reprobed with an antibody directed against the C-terminal region of human MET recognizing p45 MET, phosphorylated form of MET (Phospho-MET) and GAPDH to assess the loading. Arrowheads indicate , full-length MET, phosphorylated MET and the flag-p45 MET fragment.

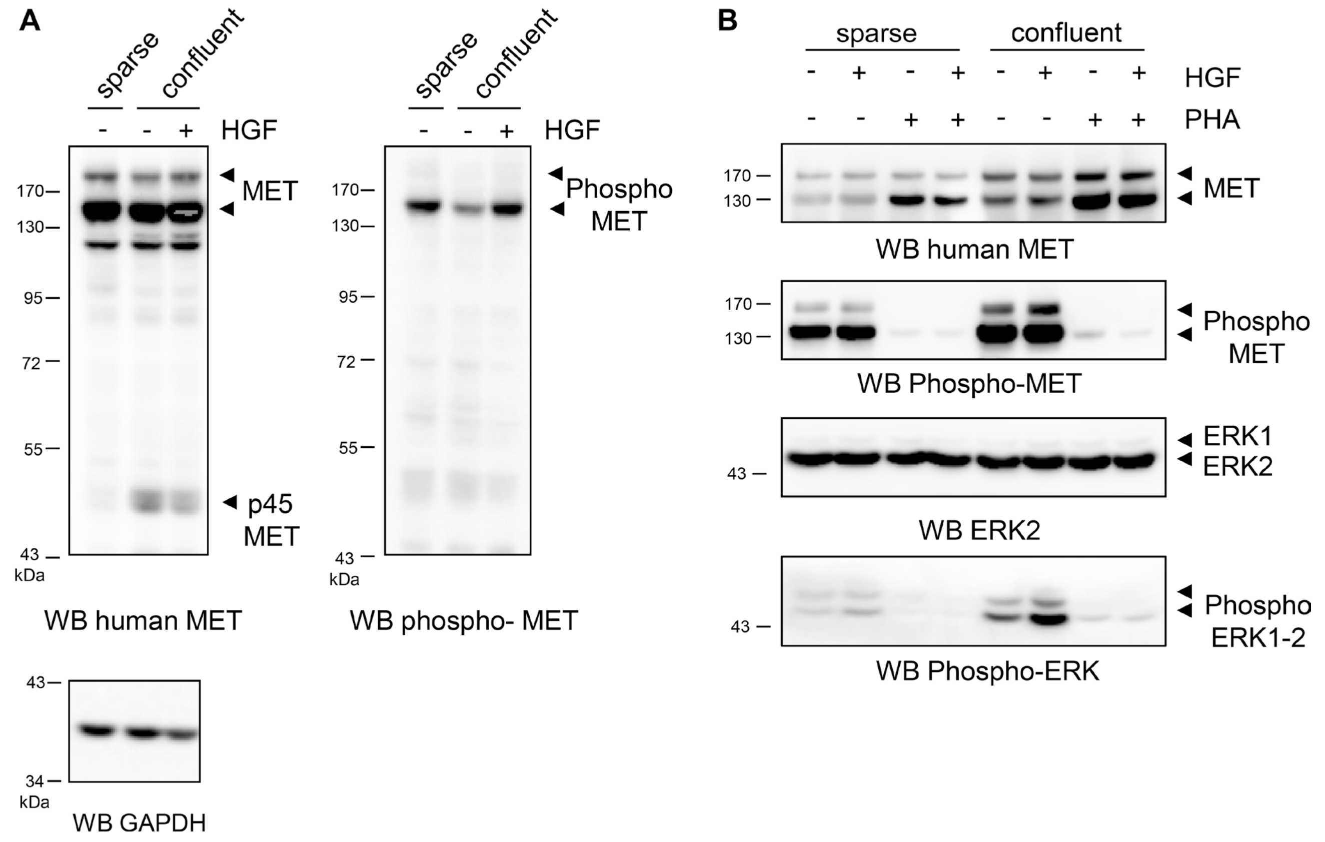

Supplementary Figure 5: (**A** and **B**) Sparse (s) and confluent NCI-H1437 cells were treated or not 1h with 0.3 mM of MET kinase inhibitor PHA-665752 (PHA) and then 10 min with 10 ng/ml HGF. (A) Cell lysates were analyzed by western blotting with an antibody directed against the human MET C-terminal region. Membrane was successively reprobed with an antibody directed against the phosphorylated form of MET (Phospho-MET) and GAPDH to assess the loading. (B) Cell lysates were analyzed by western blotting. Upper part of the filter was probed with an antibody directed against the human MET C-terminal region and reprobed with an antibody directed against its phosphorylated form (Phospho-MET). Lower part was probed with an antibody directed against ERK2 and reprobed with an antibody directed against phosphorylated form of ERK1 and 2. Arrowheads indicate total and phosphorylated MET, total and phosphorylated ERK1 and 2 and the p45 MET fragment
